# Supplementary material for: Supramolecular Tools to Improve Wound Healing and Antioxidant Properties of Abietic Acid: Biocompatible Microemulsions and Emulgels
Source: Molecules. 2022 Sep 30;27(19):6447. doi: 10.3390/molecules27196447 (PMC9572722; doi:10.3390/molecules27196447)
Supplement: Supplementary file 1 [file molecules-27-06447-s001.zip › molecules-1918422-supplementary.pdf]

Supramolecular Tools to Improve Wound Healing and Antioxidant Properties of Abietic Acid: Biocompatible Microemulsions and Emulgels

Alla Mirgorodskaya [1], Rushana Kushnazarova [1,*], Rais Pavlov [1], Farida Valeeva [1], Oxana Lenina [1], Ksenia Bushmeleva [1], Dmitry Kuryashov [2], Alexandra Vyshtakalyuk [1], Gulnara Gaynanova [1], Konstantin Petrov [1] and Lucia Zakharova [1]

[1] Arbuzov Institute of Organic and Physical Chemistry, FRC Kazan Scientific Center of RAS, Arbuzov str. 8, 420088 Kazan, Russia
[2] Kazan National Research Technological University, Karl Marx str. 68, 420015 Kazan, Russia
* Correspondence: ruwana1994@mail.ru; Tel.: +7-843-273-22-93

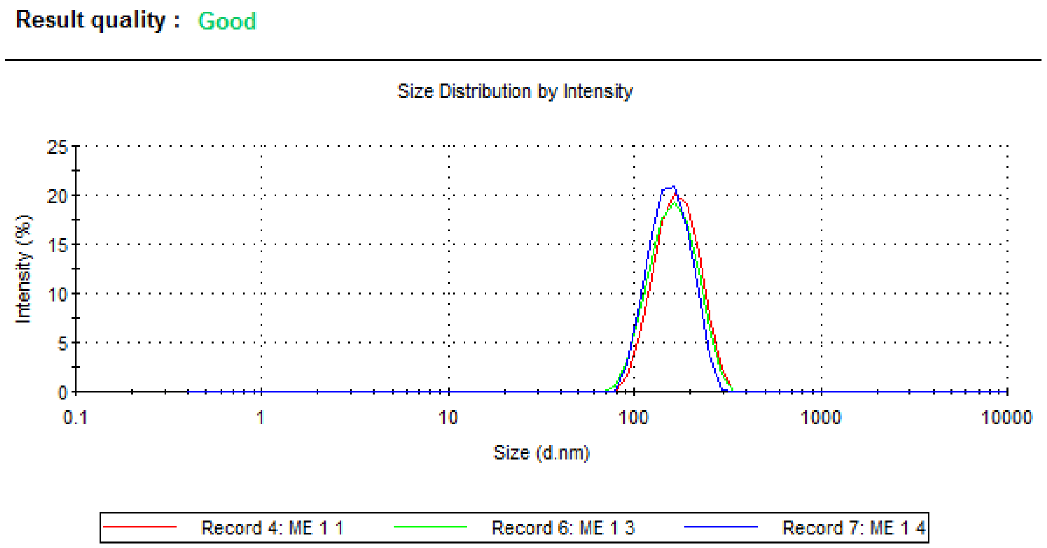

Figure S1. The DLS data: droplet size distributions in the ME 1.

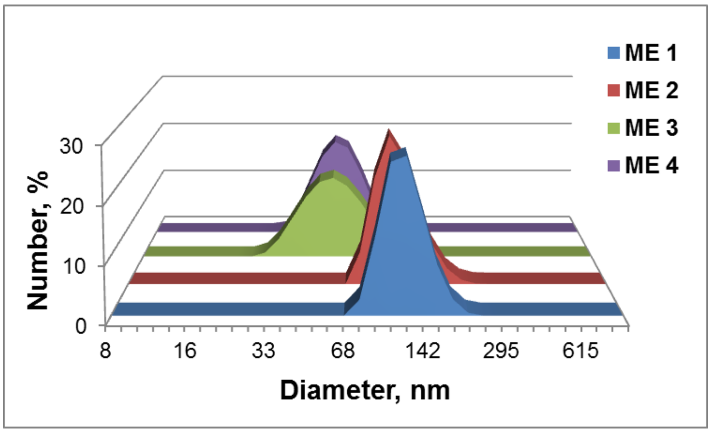

Figure S2. Droplet size distribution in different microemulsions.

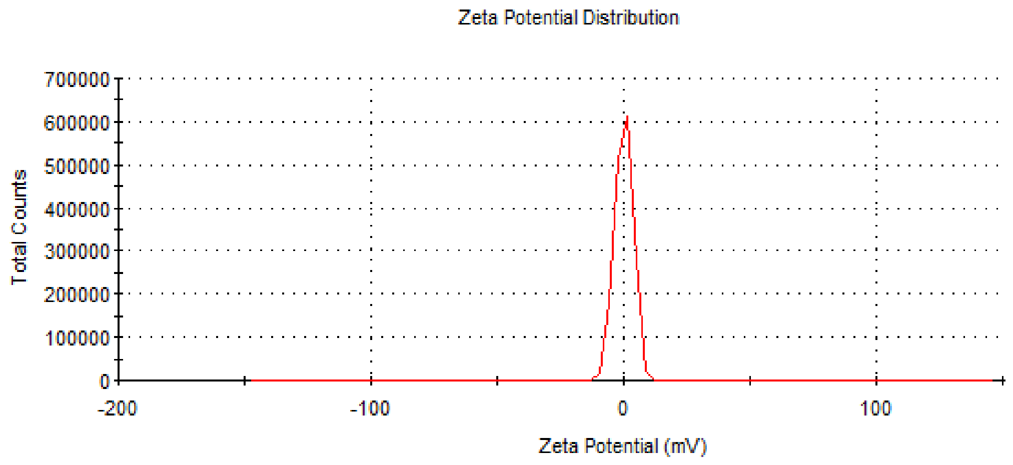

Figure S3. Zeta-potential of the ME 1 without a cationic surfactant.

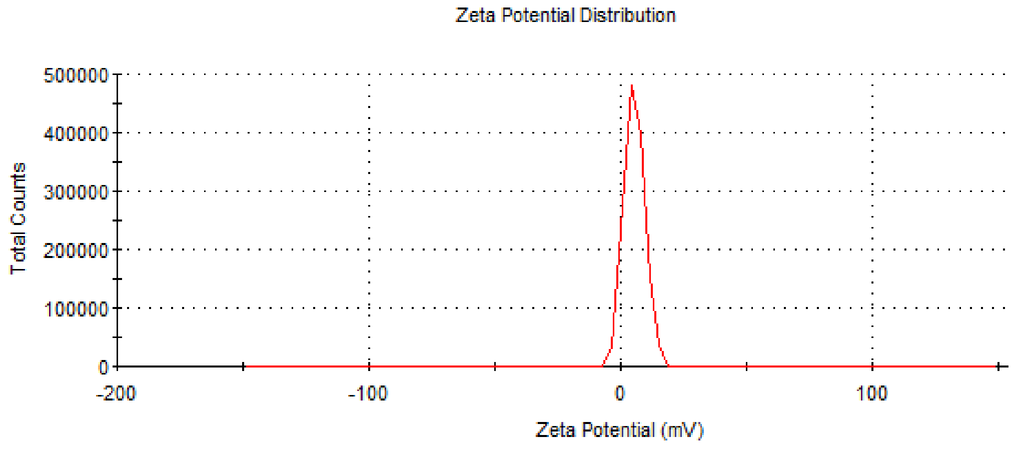

Figure S4. Zeta-potential of the ME 3 with the surfactant CB-16(Bu) (3% wt).

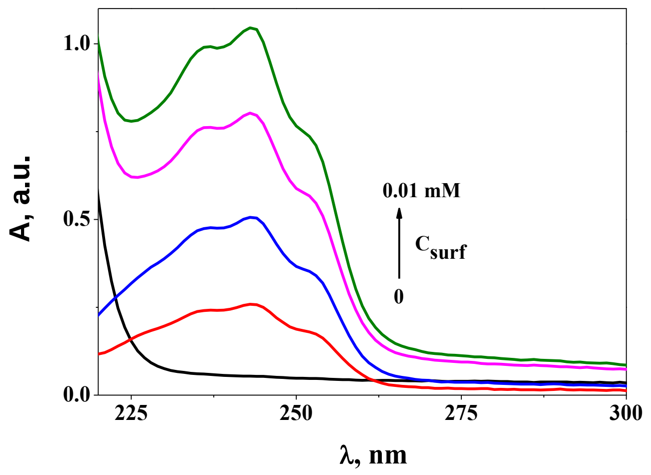

Figure S5. Spectra of saturated solutions of abietic acid recorded at various contents of surfactant CB-16(Bu).

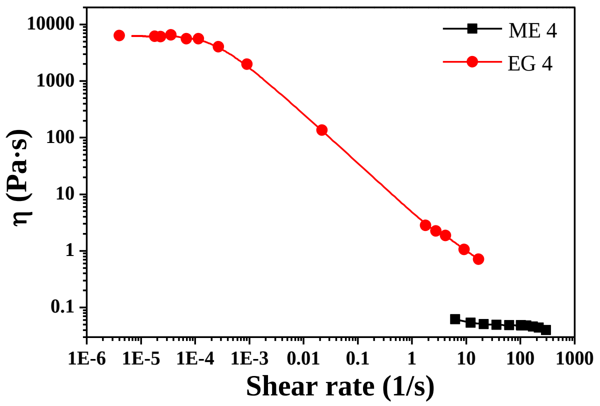

Figure S6. Apparent viscosity data for microemulsion (ME 4) and emulgel (EG 4), 25 °C.

Table S1. Position of the maximum and value of molar attenuation coefficient of abietic acid in various media.

Media	ε, l·mol^{-1}·cm^{-1}	λ_{max}, nm
Water	12,700	245
Ethanol	13,000	241
Tween 80	12,400	242
CB-16(Bu)	12,850	242
CTAB	13,100	242
PBS: Ethanol (1:1)	13,000	240
